# A Compound Fungicide Impairs Cognitive Performance in Honey Bees (*Apis mellifera*)

**DOI:** 10.3390/insects17010051

**Published:** 2025-12-30

**Authors:** Xufeng Zhang, Qian Cao, Qihang Sun, Yuting Tian, Yinyin Du, Yuan Guo

**Affiliations:** 1 College of Horticulture, Shanxi Agricultural University, Taiyuan 030001, China; zhangxf@sxau.edu.cn; 2 College of Animal Sciences, Shanxi Agricultural University, Jinzhong 030801, China; caoqian_0423@163.com (Q.C.); qihangsun1999@163.com (Q.S.); tianyuting6419@163.com (Y.T.); duyinyin032200@163.com (Y.D.)

**Keywords:** *Apis mellifera*, compound fungicide, learning and memory behavior, proboscis extension reflex, sucrose response score

## Abstract

Fungicides play a critical role in crop protection, yet their potential threats to pollinator remain a concern. This study investigated the sublethal effects of a commercial fungicide, Chunmanchun^®^ (a suspension-emulsion of 7% propiconazole and 28% carbendazim), on cognitive functions of the honey bee (*Apis mellifera*). Using the proboscis extension reflex (PER) assay, we evaluated sucrose sensitivity and olfactory learning and memory in workers exposed to the recommended field concentration (PC), along with sublethal (LD_10_) and semi-lethal (LD_50_) concentrations. Fungicide exposure significantly reduced sucrose sensitivity across all concentrations tested (0.1%, 1%, and 3%), with the strongest reductions occurring at the LD_10_ and LD_50_ levels. While olfactory associative learning was not significantly impaired, memory retention was adversely affected. Bees in the LD_50_ group showed significantly reduced PER rates at both1 h and 6 h post-training, and LD_10_ and LD_50_ groups exhibited significant memory deficits by 1 h and 6 h relative to the control. These results demonstrate that Chunmanchun^®^ impairs both sucrose responsiveness and olfactory memory in honey bees, which may impair foraging efficiency and ultimately affect colony performance. This study highlights a potential ecological risk posed by this fungicide to pollinators in agricultural environments.

## Simple Summary

This study highlights the concerning impact of the compound fungicide Chunmanchun^®^ on honey bees, which are crucial for pollination in agriculture. The research aimed to assess how different concentrations of this fungicide affect the bees’ sensitivity to sugar and their learning and memory abilities. The results indicated that exposure to Chunmanchun^®^ significantly lowered the bees’ responses to sugar solutions and impaired their ability to learn and memory. Specifically, bees exposed to sublethal and semi-lethal doses of the fungicide struggled with memory tasks hours after exposure. These findings underscore the risks that such agricultural chemicals pose to pollinator health, which is vital for crop productivity. Protecting bees from the harmful effects of fungicides not only supports their health but also ensures the sustainability of agricultural systems and food production. This research serves as an important reminder for farmers and policy-makers to consider the implications of chemical use on pollinators, advocating for practices that protect these essential insects.

## 1. Introduction

Pollinators play a crucial role in ecosystem health and agricultural productivity. They facilitate the reproduction of over 75% of flowering plants and are responsible for about one-third of global food production [1]. Honey bees are indispensable to global agriculture, enhancing the yield 0 and quality of crops by pollinating over 85% of them and 90% of fruit trees worldwide. Their pollination services hold immense economic and social value [2]. Despite this crucial role, bee populations are declining, a trend driven by agricultural intensification that causes habitat loss and increased agrochemical exposure [3,4,5,6]. Efforts to mitigate risks such as habitat loss and pesticide exposure are necessary to sustain pollinator populations and ensure continued agricultural productivity [7].

Numerous studies have demonstrated the detrimental effects of agrochemicals on bees, such as impaired cognitive function [8,9,10] and increased susceptibility to diseases [11,12,13]. Among these agrochemicals, fungicides are generally considered safe due to their low acute toxicity [14,15]. Consequently, they are frequently applied during the blooming period, directly exposing foraging bees [16,17]. Due to the potential development of pathogen resistance from prolonged use of a single fungicide, to some extent, the application of compound fungicides with different modes of action can enhance control efficacy against pathogens on pear trees and mitigate resistance development [18]. Recent research has increasingly focused on the effects of multi-site fungicides (such as chlorothalonil, mancozeb) are generally considered safer than systemic or mitotoxic agents, their impact on honey bees (*Apis mellifera*), highlighting their potential risks to bee health and behavior. Multi-site fungicides, which target multiple biochemical pathways in fungi, are often considered less harmful to non-target organisms than single-site fungicides. Unlike single-site inhibitors that target specific enzymes, multi-site fungicides act on various cellular processes, theoretically reducing resistance risk but still posing complex ecological risks. However, emerging evidence suggests that these compounds can still pose significant threats to honey bees. A ternary mixture of azoxystrobin, boscalid, and pyraclostrobin—commonly used multi-site combinations—disrupted the gut microbiota and metabolic balance of *Apis cerana cerana*. This led to morphological changes in midgut tissue and upregulation of detoxification genes [19]. Contrary to expectations, some multi-site fungicides exhibit unexpected behavioral effects. A study revealed that bees showed a mild preference for sugar syrup laced with chlorothalonil (a common multi-site protectant). This attraction might lead bees to consume contaminated nectar or pollen, increasing their overall chemical load [20].

Although propiconazole is classified as a low-toxicity fungicide according to the pesticide toxicity classification standards in my country, it does not exert direct toxic effects on bees at field dose levels. However, it still negatively impacts bee health. Research indicates that propiconazole can disrupt the symbiotic relationship between bees and fungi and delay larval development, with the extent of these effects varying by bee species [21]. The presence of azole fungicides may enhance the toxicity of insecticides due to their competition with insecticides for cytochrome P450 (CYP450) enzymes, which delays the metabolic breakdown of these insecticides [22]. Experiments with chlorothalonil and propiconazole have demonstrated both non-lethal and lethal effects on bee colonies. Although worker bees exhibited increased pollen-feeding behavior and showed a higher rate of natural queen replacement post-exposure, there were detrimental effects such as decreased egg production, atrophy of hypopharyngeal glands, and increased susceptibility to pathogens within treated colonies [23]. Field studies revealed propiconazole residues in flowers, pollen, and in Italian workers of honey bees following application prior to crop flowering. These exposures were associated with shorter worker lifespans and enlarged hypopharyngeal acini in 5-day-old nurse bees [24]. Research into pesticide residues in pollen samples from various regions of my country identified carbendazim as the most frequently detected fungicide, with a detection rate of 77.1% [25]. Carbendazim also exhibited the highest detection frequency in pesticide residue testing of bee products [26]. Given that bees primarily rely on pollen for protein, the risk of exposure to carbendazim contaminants in their environment is significantly heightened. Although classified as a low-toxicity fungicide, the transhumant beekeeping practices in China may introduce pollinators from contaminated farms to new sites, thus increasing the risk of secondary or multiple contaminations that threaten bee survival. Studies have shown that exposure to carbendazim significantly inhibits the expression of crucial antimicrobial peptide genes, hymenoptaecin and apidaecin, which serve as indicators of bees’ immune responses. Additionally, field residues of carbendazim may interfere with detoxification processes mediated by the CYP450 enzyme system, weakening colonies’ resilience to environmental stressors [27]. Furthermore, exposure to carbendazim in Italian bees correlates with downregulation of neuroregulatory genes, indicating a potential neurotoxic effect on bees’ nervous systems [28]. In recent years, the compound fungicide Chunmanchun^®^-a suspension-emulsion of 7% propiconazole and 28% carbendazim (Chunmanchun^®^; Zhaoyuan Sanlian Chemical Co., Ltd., Zhaoyuan, China) has been widely and repeatedly applied during the flowering period of pear trees in agricultural production in Shanxi Province of China. Its use aims to control pear diseases and enhance calyx removal in young fruits. However, application during periods of active honey bee foraging results in direct exposure of worker bees to the fungicide mixture. While these low-toxicity fungicides at recommended doses do not cause immediate bee mortality, a growing body of research indicates they pose significant sublethal threats. Exposure to such fungicides has been shown to impair bee foraging behavior [29,30,31], reduce learning capacity [32,33], and decrease pollination efficiency. Furthermore, it can disrupt larval development [34,35,36], weaken immune defenses [37,38], and ultimately lower survival rates [23,39]. The latest research of compound fungicide Chunmanchun^®^ suggests that sublethal effects of gut disturbance and cognitive impairments for honey bees [40]. However, field-realistic risks are understudied, and controlled laboratory experiments are needed as a first step.

In this study, experiments were conducted to examine the effects of compound fungicides at different concentrations: Field recommended concentrations (PC), Sublethal dose (LD_10_), and Semilethal dose (LD_50_) on sucrose sensitivity of honey bees, the behavior of associative learning and memory by proboscis extension reflex (PER), associative learning and memory. Our results will provide a theoretical basis for the rational use of compound fungicides in agricultural production, and also provide a vital reference for continuously improving the risk assessment system of pesticides on pollinators.

## 2. Materials and Methods

### 2.1. Tested Bee Colonies

Three colonies of *Apis mellifera* (hereafter referred to as ‘bees’) were obtained from the Experimental Apiary of the College of Horticulture, Shanxi Agricultural University. They had not been exposed to any pesticides before the experiment and were managed and fed uniformly during the experiment. Forager bees, which were collecting pollen on their hind legs, were collected at the hive entrance using tweezers as test bees and placed in a homemade feeding box. The feeding box (23 cm × 9.6 cm × 5.3 cm) was designed with a transparent plastic pull door at the top, ventilation holes were set around and at the top, and feeding strips were attached at the bottom.

### 2.2. Reagents for Experiment

A compound fungicide containing 7% propiconazole and 28% carbendazim suspension-emulsion (Chunmanchun^®^, Zhaoyuan Sanlian Chemical Co., Ltd., Zhaoyuan, China) and 50% (*w*/*v*) sucrose (purchased from Jiangsu Baimei Sugar Industry Co., Ltd., Huaian, China) solution was used to dilute the stock solution to prepare the final feeding solution. Citral (purchased from Shanghai Aladdin Biotechnology Co., Ltd., Shanghai, China).

### 2.3. Treatment of Bees

The captured foragers were placed in the feeding boxes, 50 bees per box, in an artificial climate incubator at (33 ± 2) °C with a relative humidity of 50% ± 10% and kept in the dark. In the control group and each treatment group, bees in the feeding boxes were only fed with 50% (*w*/*v*) sugar solution on the first day to adapt to the artificial culture environment. On the second day, drug administration and feeding were carried out. The three different concentrations of Chunmanchun^®^ were calculated experimentally and also referenced data from our previously published studies [40]. LD_50_ group: semi-lethal dose 1.011 g/L of compound fungicide sugar solution; LD_10_ group: sublethal dose 0.308 g/L of compound fungicide sugar solution; PC group: the field recommended dose 0.159 g/L of compound fungicide sugar solution; Control group, bees were fed with 50% (*w*/*v*) sugar solution, and bees in each group were allowed to feed ad libitum. After 24 h of treatment, the bees were subjected to a gradient concentration sucrose sensitivity test and an associative odor learning test, and each test was repeated three times.

### 2.4. Sucrose Sensitivity Test

Bees were briefly anesthetized with CO_2_ (10 s), then harnessed in plastic tubes (6 mm diameter) that immobilized the body but left the head and forelegs exposed. The straw was inserted from the side with an insect needle and passed through the nodules on the bee’s chest and abdomen, effectively trapping the bee to prevent it from crawling out. The plastic pipette was then fixed to a wooden board and placed in a constant temperature incubator at 30 °C and 50% relative humidity in the dark for 2 h. After the bees recovered, normal and active bees were selected for the experiment. For the PER assay, bees were harnessed individually according to established methods to restrict body movement during training and testing [41].

To facilitate the adaptation of bees to the culture environment, all experimental groups were provided with a 50% sucrose solution on the first day. On the second day, bees in each treatment group were allowed to consume a compound fungicide solution at varying concentrations for 24 h to minimize potential experimental error. A sucrose solution with progressively increasing gradient concentrations was utilized to assess the response of test bees to varying concentrations of sucrose. Different weights of sucrose were accurately measured and dissolved in distilled water using an analytical balance. Sucrose solutions with gradient concentrations of 0.1%, 0.3%, 1%, 3%, 10%, and 30% (*w*/*v*) were prepared and placed into 10 mL centrifuge tubes for subsequent use. Fixed bees were examined, and inactive or dead individuals were removed. A cotton swab, moistened with distilled water, was employed to stimulate the antennae of active bees. Bees exhibiting a PER were fed distilled water multiple times until they ceased extending their proboscises. After stimulation with different concentrations of sucrose solution, distilled water was used to counteract the sensitizing effect of sucrose solution on the honey bee proboscis. The stimulation interval was set at two minutes, and the response of honey bees to stimulation with diverse sucrose solution concentrations was meticulously documented. A positive response was designated as “+”, while a negative response was assigned as “−”. In the absence of PER to sucrose stimulation at any of the aforementioned concentrations, the bee was excluded from subsequent analysis. The sucrose response score (SRS) was determined by the summation of the number of PER exhibited by each bee in response to sucrose concentrations ranging from 0.1% to 30% (*w*/*v*) [42]. The SRS ranged from 0 to 6, with SRS = 0 indicating that individual bees did not respond to all tested sucrose concentrations except 50% (*w*/*v*) sucrose solution, and SRS = 6 indicating that bees showed a proboscis response to sucrose concentrations ranging from 0.1% to 30% (*w*/*v*).

### 2.5. Odor-Associative Learning and Memory Test

The fixation of bees was the same as the above experiment. Prior to formal odor stimulation, an empty syringe was used to blow at the tip of the bees’ fixed antennae (without making contact with the antennae) for 4 s to determine whether the bees would produce a PER. The individual bees that exhibited a PER were then replaced in order to eliminate the potential influence of airflow on the bees. Consequently, in the absence of odor stimulation during the test, no PER occurred in any of the bees tested. The bees were immobilized and then individually fed with cotton swabs dipped in a sucrose solution (50% *w*/*v*) for 3 s. This procedure was performed 0.5 h prior to the commencement of the test. Any individual bee that did not extend its proboscis during the feeding process also had to be replaced immediately before subsequent PER tests.

The syringe, containing citral filter paper, was directed towards the uppermost extremity of the antennae of fixed bees for a period of four seconds, during which time constant blowing was performed. At the fourth second of the blowing process, a cotton swab dipped in 50% (*w*/*v*) sucrose solution was used to challenge the bee’s antennae for four seconds. If a PER to citral was observed in the first three seconds, the response was recorded as a response to the conditioned stimulus (CS) (+) or as a response to the unconditioned stimulus (US) (−) if the proboscis was only extended to 50% (*w*/*v*) sucrose solution in the last 3 s of the current round of trials. The temporal separation between the CS and US stimuli was 1 s, and the data pertaining to the bee responses were recorded concurrently. The interval between each test round was five minutes. The PER of bees to odor was observed and recorded. Following each trial, the bees were ventilated to eliminate the potential impact of odors from the preceding trials. Individual bees from each treatment were subjected to six training trials to establish the association between the stimulus odor and the sucrose reward.

Following the completion of the associative learning phase, the bees were returned to the artificial climate incubator and maintained in conditions of darkness. The memory test was conducted after an interval of 1 h, 3 h and 6 h, respectively. Individuals exhibiting a PER to citral odor were designated as (+), while those demonstrating an absence of PER were designated as (−). The latter group was considered to exhibit memory behavior.

### 2.6. Data Analysis

The PER of bees in each group to 0.1% to 30% (*w*/*v*) sucrose gradient concentration was recorded, and proboscis extension response of bees in each group to 6 times of odor associative learning and 1 h, 3 h and 6 h memory tests were recorded. Repeated measures ANOVA (SPSS 23.0) was used to analyze the differences in response to gradient sucrose concentration, associative learning behavior and memory behavior between CK, PC, LD_10_ and LD_50_ group, *n* = 240, with 3 independent replicates of 20 bees per group each. To further analyze the differences in the responses of bees to gradient sucrose water concentrations among the groups, sucrose response scores (SRS) were calculated for each group, SRS was between 0 and 6, and the overall fluctuation of SRS in CK, PC, LD_10_, and LD_50_ groups was analyzed by Kruskal–Wallis H test, *n* = 240, with 3 independent replicates of 20 bees per group each, multiple-comparison corrections Bonferroni were applied.

## 3. Results

### 3.1. Compound Fungicides Affect the Response of Bees to Sucrose

**Figure 1:**
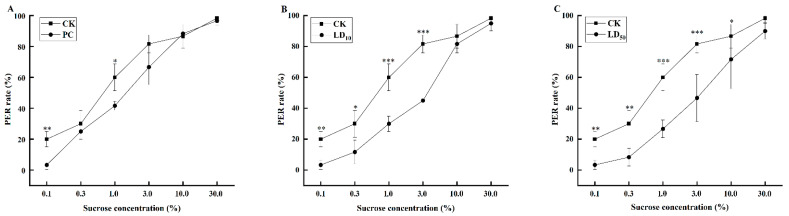
Effects of Chunmanchun^®^ on the sucrose concentration response of foragers of *Apis mellifera*. (**A**) The comparison between PC and CK. (**B**) The comparison between LD_10_ and CK. (**C**) The comparison between LD_50_ and CK. CK = control, PC = exposure to Chunmanchun^®^ at the field-recommended concentration of 0.159 g/L, LD_10_ = exposure to Chunmanchun^®^ at the sublethal concentration of 0.308 g/L. LD_50_ = exposure to Chunmanchun^®^ at the sublethal concentration of 1.011 g/L. * represents *p* < 0.05; ** represents *p* < 0.01; and *** represents *p* < 0.001. *n* = 240, with 3 independent replicates of 20 bees per group each, the error bars were standard deviation.

The PER rate of the bees was observed and recorded in a sucrose solution with concentrations ranging from 0.1% to 30%, and the results are presented in Figure 1, the original data was shown in Supplemental Table S1.

The results demonstrated a positive correlation between the concentration of sucrose solution and PER rate, with higher sucrose concentrations elicited higher PER rates. However, the PER rate of bees in the compound fungicide treatment groups was lower than that in the control group. When bees were stimulated with 0.1% sucrose solution, the PER rate of bees in each treatment group was significantly lower than that in CK group (PC, LD_10_ and LD_50_ group: χ^2^ = 8.086, *p* = 0.004). When the sucrose concentration increased to 0.3%, the PER rate of bees showed a concentration-dependent decrease. The PER rate of bees in LD_10_ group was significantly lower than that in CK group (χ^2^ = 6.114, *p* = 0.013), and the PER rate of bees in LD_50_ group was significantly lower than that in CK group (χ^2^ = 9.090, *p* = 0.003), and the PER rate of bees in PC group was not significant (χ^2^ = 0.376, *p* = 0.540). When the sucrose concentration increased to 1%, the PER rate of bees in the PC group was significantly lower than that in the CK group (χ^2^ = 4.034, *p* = 0.045), and the PER rate of bees in the LD_10_ and LD_50_ groups was significantly lower than that in the CK group (LD_10_ group: χ^2^ = 10.909, *p* < 0.001; LD_50_ group: χ^2^ = 13.575, *p* < 0.001). When the sucrose concentration increased to 3%, the PER rate of bees in the PC group was lower than that in the CK group, but the difference was not significant (χ^2^ = 3.523, *p* = 0.061). The PER rate of bees in the LD_10_ and LD_50_ groups were significantly lower than that in the CK group (LD_10_ group: χ^2^ = 17.368, *p* < 0.001; LD_50_ group: χ^2^ = 15.983, *p* < 0.001). When the sucrose concentration was 10%, the PER rate of bees in the PC group had no significant compared with CK (χ^2^ = 0.076, *p* = 0.783), the PER rate of bees in the LD_10_ group had no significant compared with CK (χ^2^ = 0.453, *p* = 0.563), the PER rate of bees in the LD_50_ group was significantly lower than that in the CK group (χ^2^ = 4.093; *p* = 0.043). When the sucrose concentration was 30%, the PER rate of bees in the PC group had no significant compared with CK (χ^2^ = 0.342, *p* = 0.559), the PER rate of bees in the LD_10_ group had no significant compared with CK (χ^2^ = 0.259, *p* = 0.611), the PER rate in LD_50_ group was also lower than that in CK group, but the difference was not significant CK (χ^2^ = 2.427, *p* = 0.119). To further study the effect of compound fungicides on honey bee sucrose sensitivity after exposure to different concentrations, we calculated sucrose response scores (SRS) for individual bees. The antennae of bees that did not respond to any of the six sucrose gradients were stimulated again with a cotton swab stained with 50% sucrose solution to observe whether they extended their proboscis. Then the SRS of individual bees in each group were obtained, and the sensitivity of individual bees in each group to sucrose solution was further evaluated.

**Figure 2:**
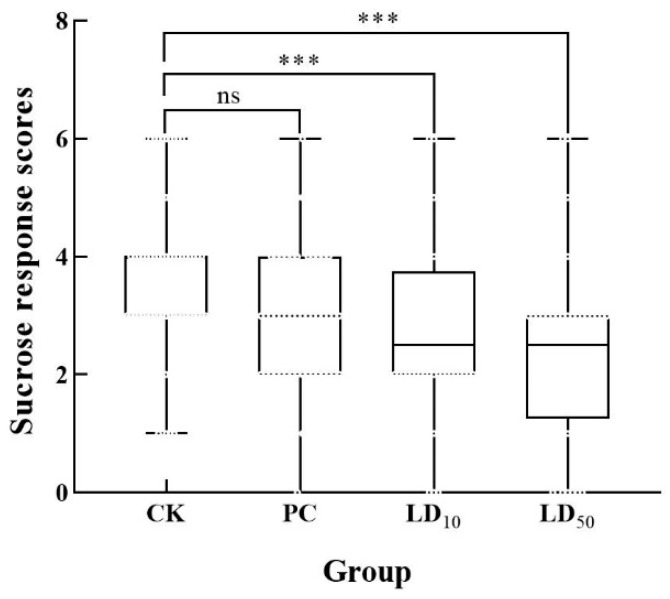
Effects of Chunmanchun^®^ on the sucrose response scores (SRS) of foragers of *Apis mellifera*. There was no significant difference in SRS between the PC and CK group; the SRS of honey bees in the LD_10_ and LD_50_ group were significantly lower than those in CK group. CK = control, PC = exposure to Chunmanchun^®^ at the field-recommended dose of 0.159 g/L, LD_10_ = exposure to Chunmanchun^®^ at the sublethal dose of 0.308 g/L. LD_50_ = exposure to Chunmanchun^®^ at the semilethal dose of 1.011 g/L. Kruskal–Wallis H test, Z = 29.633, *p* < 0.001. *** represents *p* < 0.001. ns represents *p* > 0.05. *n* = 240, with 3 independent replicates of 20 bees per group each, the error bars were standard deviation.

There was no significant difference in SRS between bees in PC and CK groups (Figure 2) (*p*_(*Bonferroni*)_ = 0.206). The SRS of bees in LD_10_ and LD_50_ groups were significantly lower than those in CK group (Kruskal–Wallis H test, Z = 29.633, *p*_(*Bonferroni*)_ < 0.001; PC versus CK: *p*_(*Bonferroni*)_ = 0.206; LD_10_ versus CK: *p*_(*Bonferroni*)_ < 0.001; LD_50_ versus CK: *p*_(*Bonferroni*)_ < 0.001).

### 3.2. Effects of Chunmanchun^®^ on the Learning Behavior of Bees

**Figure 3:**
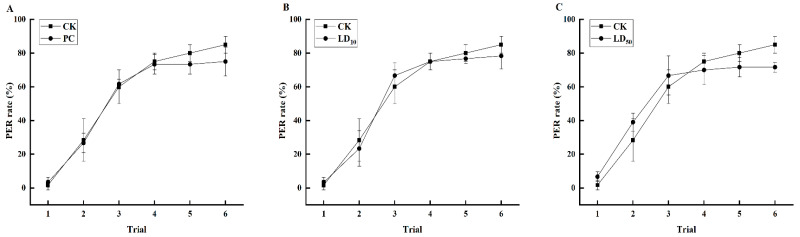
Effects of Chunmanchun^®^ on the learning behavior of *Apis mellifera* foragers. (**A**) The proportion of honey bees exhibiting a proboscis extension reflex (PER) during six successive classic conditioning trials was not significantly different between the Chunmanchun^®^-treated (PC) and control (CK) groups. (**B**) The proportion of honey bees exhibiting a PER during six successive classic conditioning trials was not significantly different between the Chunmanchun^®^-treated (LD_10_) and control (CK) groups. (**C**) The proportion of honey bees exhibiting a PER during six successive classic conditioning trials was not significantly different between the Chunmanchun^®^-treated (LD_50_) and control (CK) groups. CK = control, PC = exposure to Chunmanchun^®^ at the field-recommended concentration of 0.159 g/L, LD_10_ = exposure to Chunmanchun^®^ at the sublethal concentration of 0.308 g/L. LD_50_ = exposure to Chunmanchun^®^ at the sublethal concentration of 1.011 g/L. (Repeated measures ANOVA, *n* = 240, with 3 independent replicates of 20 bees per group each, the error bars were standard deviation.)

As demonstrated in Figure 3, the PER rate increased in each group of bees following training with citral odor paired with sucrose reward during the six associative learning sessions. In the initial four associative learning sessions, the PER rate of the PC group and the CK group exhibited a tendency to be similar, the original data was shown in Supplemental Table S2. However, during the fifth and sixth associative learning sessions, the PER rate of bees in the PC group exhibited a gradual decline compared to the CK group, though these differences did not attain statistical significance (*F*_(5,114)_ = 5.769, *p* = 0.138). The PER rates in the LD_10_ group and control (CK) group were comparable in both the first and fourth associative learning sessions. In the second session, the PER rate for the LD_10_ group was lower than that of the CK group; however, this difference was not statistically significant. Conversely, in the third associative learning session, the PER rate for the LD_10_ group exceeded that of the CK group, yet the difference also lacked statistical significance. In the fifth and sixth sessions, the PER rates in the LD_10_ group progressively declined compared to the CK group, with the differences remaining statistically insignificant (*F*_(5,114)_ = 4.000, *p* = 0.184). In the first three associative learning times of LD_50_ group, the PER rate of bees in was higher than that in CK group, but the difference was not significant. However, in the last three instances of associative learning, the PER rate of bees in the LD_50_ group was gradually lower than that of bees in the CK group, and the difference was also not significant (*F*_(5,114)_ = 0.027, *p* = 0.885).

### 3.3. Effects of Chunmanchun^®^ on the Memory Behavior of Bees

**Figure 4:**
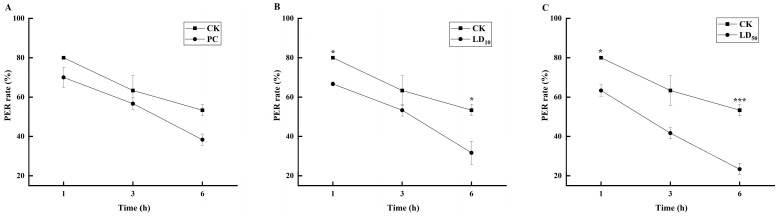
Effects of Chunmanchun^®^ on the memory behavior of *Apis mellifera* foragers. (**A**) Recall tests between PC and CK group. (**B**) Recall tests between LD_10_ and CK group. (**C**) Recall tests between LD_50_ and CK group. CK = control, PC = exposure to Chunmanchun^®^ at the field-recommended concentration of 0.159 g/L, LD_10_ = exposure to Chunmanchun^®^ at the sublethal concentration of 0.308 g/L. LD_50_ = exposure to Chunmanchun^®^ at the sublethal concentration of 1.011 g/L. Recall tests showed a consistently lower performance of different treatments than CK group, the difference was significant after 1 h and 6 h between LD_10_ and CK group, the difference was significant after one hour, and extremely significant after six hours between LD_50_ and CK group (repeated measures ANOVA, *n* = 240, with 3 independent replicates of 20 bees per group each, the error bars were standard deviation, * represents *p* < 0.05, *** represents *p* < 0.001).

The memory results of bees in each group at 1 h, 3 h and 6 h are shown in Figure 4. There had no significance between PC and CK group at the time point of 1 h (*F*_(5,114)_ = 12.000, *p* = 0.074), 3 h (*F*_(5,114)_ = 4.000, *p* = 0.184), and 6 h (*F*_(5,114)_ = 2.695, *p* = 0.103). There had also no significance between LD_10_ and CK group at the time point of 3 h (*F*_(5,114)_ = 4.000, *p* = 0.184), but there was significant difference at the time point of 1 h (*F*_(5,114)_ = 64.000, *p* = 0.015) and 6 h (*F*_(5,114)_ = 42.250, *p* = 0.023). Between LD_50_ and CK group, there was no significance at 3 h (*F*_(5,114)_ = 16.000, *p* = 0.057), but there was a significant difference at 1 h (*F*_(5,114)_ = 10.000, *p* = 0.010) and extremely significant difference at 6 h (*F*_(5,114)_ = 12.234, *p* < 0.001). Memory performance declined over time in all groups, but the reduction was more pronounced in fungicide-exposed bees. LD_50_ bees showed significant deficits at 1 h and 6 h, and all treated groups exhibited significant impairment by 1 h and 6 h. The original data was shown in Supplemental Table S2.

## 4. Discussion

In contemporary agricultural practices, the use of fungicides to control fungal diseases in crops plays a vital role in ensuring both yield and quality [43]. Propiconazole and carbendazim are widely employed as low-toxicity fungicides, particularly during the flowering stage of crop development. However, it is essential to recognize that bees are often exposed to these fungicides due to their interactions with treated crops. Although the recommended doses typically do not result in acute mortality among bees, they may still pose significant threats to their health. Carbendazim, a heterocyclic fungicide derived from natural sources, disrupts the synthesis of deoxyribonucleotides in fungi and interferes with mitotic processes, thereby exerting antifungal effects and effectively controlling crop diseases. It is a commonly used fungicide in managing diseases affecting field crops, vegetables, fruit trees, and cash crops [44,45]. Nevertheless, even minor alterations in the structure of spindle microtubule proteins may impact the binding of carbendazim, which could predispose pathogens to develop drug resistance [46]. Propiconazole, an emerging fungicide, has been demonstrated to cooperate with 14-α demethylase (CYP51) to inhibit the metabolic pathway responsible for ergosterol synthesis in harmful bacteria. This collaborative mechanism disrupts the integrity of bacterial cell membranes, leading to cell death and contributing to sterile conditions that suppress bacterial pathogens [47]. As an endogenously occurring triazole fungicide, propiconazole exerts both protective and therapeutic effects, making it particularly effective in cash crop cultivation and categorizing it within the sterol inhibitor group. The combination of propiconazole and carbendazim has been shown to enhance the effectiveness of crop protection against pathogens while also mitigating the development of drug resistance. For honey bees, precise navigation in search of nectar and pollen, as well as accurate positioning of their nests, is crucial for the physiological well-being of both individuals and colonies [48]. These activities are fundamentally linked to honey bees’ response, learning, and memory behaviors related to sucrose. Ensuring the health of pollinators while effectively managing crop diseases and insect infestations is a key component of sustainable agricultural development. In this study, we investigated the effects of a mixture of 35% propiconazole and carbendazim at recommended, sublethal, and semi-lethal concentrations on the sucrose sensitivity, learning, and memory behaviors of honey bees.

The results of this study suggest that exposure to compound fungicides adversely affected the sucrose sensitivity, learning, and memory abilities of honey bees, with the severity of these effects increasing in a concentration-dependent manner. These impairments may translate into reduced foraging efficiency; however, colony-level impacts require further study using colony-based experiments. The results align with observed declines in bee populations following fungicide applications in intensively managed commercial orchards [49,50], highlighting concerns regarding the sublethal effects of pesticide mixtures that bees are routinely exposed to in agricultural settings [51,52,53]. Notably, exposure to the recommended field dose of the compound fungicide did not significantly impair sucrose sensitivity or cognitive behaviors in honey bees, consistent with the documented low acute toxicity of fungicides of Sterol Biosynthesis Inhibitors to bees [50,54,55,56]. In our previous study, exposure to the recommended field dose of Chunmanchun^®^ also did not impair the learning behavior of honey bees [40], which is consistent with the results of our study. The learning behavior of the LD_10_ and LD_50_ groups was also not impaired. However, the sublethal and semi-lethal doses of compound fungicide Chunmanchun^®^ significantly impaired the memory function of 1 h and 6 h; this trend is consistent with the trend observed in our previous study [40].

Most fungicides exhibit extended residual periods in water and soil [57]. Our results suggest that when fungicide accumulation in the bees’ environment reaches a critical threshold, it may pose substantial and unpredictable risks. Moreover, under natural conditions, bees are more frequently exposed to pesticide mixtures rather than individual compounds. Such mixtures can interact synergistically, antagonistically, or additively [58]. In studies assessing combined toxicity in honey bees, 72% of 92 pesticide formulations exhibited synergistic effects—where the combined toxicity exceeded that of individual components—while 17% showed additive effects [59]. Therefore, further investigation into the impact of compound fungicides on bee foraging behavior is warranted.

As demonstrated by certain studies, exposure to propiconazole has been shown to have the potential to induce aberrant behaviors in *Apis mellifera* workers, including hyperactivity and impaired coordination. However, it has been observed that as the action time of propiconazole is extended, these aberrant behaviors gradually dissipate [60]. When *Apis mellifera* workers were exposed to different concentrations of carbendazim, the higher the concentration, the lower the pollen intake of workers, indicating that carbendazim has a certain repellent effect on bees [18]. Our study showed that honey bee sensitivity to sucrose and memory behavior decreased gradually with the increase in compound fungicide concentration, which was significantly different from the control group. Co-application with fungicides results in a significant decrease in colony collection capacity [61,62]. When *Apis mellifera* were exposed to different concentrations of the compound fungicide Pristine^®^: A combination of 25.2% Boscalid and 12.8% Pyraclostrobin not only significantly decreased the learning and metabolic abilities of worker bees [9,10], and it also caused individual worker bees exposed to the compound fungicide in the larval stage to prefer pollen collection in the forager stage [30]. In addition to laboratory experiments, a large number of field experiments also showed that Thiophanate methyl and chlorothalonil fungicides had adverse effects on the behavior of *Apis mellifera*. As the number of field applications of thiophanate methyl and chlorothalonil increased, the frequency of visits by bees to flowers exposed to these chemicals decreased. Furthermore, the application of fungicides such as Azoxystrobin, Fenethanil, and Prothioconazole in the field has been observed to result in the repulsion of bees, thereby reducing pollination efficiency [63]. Honey bees, as location foragers, rely heavily on learning and memory behaviors, which are essential for optimizing food resources and ensuring population reproduction [64,65]. Therefore, when these cognitive abilities of bees are impaired to varying degrees, their survival is at significant risk [65]. Our latest research on the compound fungicide Chunmanchun^®^ suggested that it could negatively impact bee health beyond acute individual-level toxicity [40].

In this study, we investigated the effects of a compound fungicide at recommended, sublethal, and semi-lethal concentrations on the sucrose sensitivity, learning, and memory of *Apis mellifera* foragers. The aim was to provide scientific guidance for the rational application of this commonly used fungicide during the flowering period of fruit trees. As eusocial insects, honey bees exhibit colony health that is highly dependent on the chemical exposure experienced by individual members [66]. Honey bees rely on their sucrose sensitivity to identify and evaluate food sources. Impaired sucrose sensitivity can lead to difficulties in locating and foraging for nectar-rich flowers [67]. This lack of efficiency can result in decreased food collection, which is crucial for the survival of the colony [68]. Impairments in memory may affect a bee’s ability to accurately recall and communicate the location of successful foraging trips [69,70]. This may lead to reduced recruitment of foragers, as fewer bees are informed about available food resources [61]. Furthermore, reduced foraging may lead to insufficient food supplies for the brood, resulting in poor brood provisioning [71]. This can affect colony growth and reproductive success since the health of larval bees is directly linked to the quality and quantity of food provided. Given the complex demographic composition of a honey bee colony, further research is needed to evaluate how compound fungicides affect bees of different ages, castes, and developmental stages. Furthermore, the considerable economic importance of bees and hive products—such as royal jelly, propolis, bee pollen, and honey—which are closely linked to human health, must be emphasized. Therefore, the prudent use of fungicides in agricultural settings adjacent to apiaries is essential to minimize pesticide residues and their potential effects on bee health and the safety of bee-derived products.

## Data Availability

The original contributions presented in this study are included in the article. Further inquiries can be directed to the corresponding author.

## Supplementary Materials

The following supporting information can be downloaded at https://www.mdpi.com/article/10.3390/insects17010051/s1, Table S1: Effects of Chunmanchun^®^ on the sucrose concentration response of foragers of *Apis mellifera*. Table S2: Effects of the compound fungicide Chunmanchun^®^ on learning and memory ability of *Apis mellifera* (*n* = 20, 3 replicates for each group, extending the proboscis record as 1, not extending the proboscis record as 0).

## Abbreviations

The following abbreviations are used in this manuscript:

PER	Proboscis extension reflex
SRS	Sucrose response score
